# Establishment of a bleeding risk model for low-molecular-weight heparin in cancer-associated venous thromboembolism: a single-center retrospective study

**DOI:** 10.3389/fphar.2025.1677891

**Published:** 2025-09-29

**Authors:** Ziyu Zhang, Houfeng Zhou, Changyu Ren, Lankai Liao

**Affiliations:** ^1^ Department of Pharmacy, Chengdu Fifth People’s Hospital, Chengdu, Sichuan, China; ^2^ Department of Emergency Medicine, Chengdu Fifth People’s Hospital, Chengdu, Sichuan, China

**Keywords:** venous thromboembolism (VTE), low-molecular-weight heparin (LMWH), cancer, bleeding risk model, HAS-BLED score

## Abstract

**Objectives:**

To establish a bleeding prediction model for the use of low molecular weight heparin anticoagulation in cancer patients, aiming to help medical staff to individually evaluate the timing of low molecular weight heparin use in cancer patients.

**Methods:**

This retrospective cohort study enrolled 731 cancer patients (aged ≥18 years) from January to December 2021 receiving LMWH for venous thromboe-mbolism (VTE) prophylaxis at a tertiary general hospital in Southwest China. Participants were stratified into bleeding (n = 19) and non-bleeding (n = 712) cohorts based on ISTH-defined clinical outcomes. Risk factors identified through multivariablebinary logistic regression, with subsequent development and internal validation performed using R software (version 4.3.2).

**Results:**

Univariate analysis of bleeding risk factors revealed statistically significant differences (P < 0.05) in body weight, nonsteroidal anti-inflammatory drug (NSAIDs) use, LMWH dosage, prothrombin time (PT), creatinine clearance, platelet count, Padua score, and HAS-BLED bleeding risk score. Based on clinical relevance, the final model incorporated LMWH dosage, platelet count, and HAS-BLED bleeding risk score as assessment items. The model demonstrated excellent predictive ability for bleeding events, with an ROC-AUC (95% CI) of 0.90 (0.82–0.97). The model showed good discrimination (Hosmer-Lemeshow, P = 0.854 > 0.05) and decision making capability, with strong diagnostic performance (accuracy: 0.83, sensitivity: 0.83, specificity: 0.79, positive predictive value: 0.99). The model had a low probability of missed diagnoses and high sensitivity and specificity.

**Conclusion:**

This study developed an optimized bleeding risk prediction model by enhancing the HAS-BLED score through the integration of three key variables: HAS-BLED score, LMWH dosage, and platelet count, demonstrating a robust application prospect for anticoagulation management in cancer patients.

## 1 Introduction

Venous thromboembolism (VTE), which includes deep vein thrombosis (DVT) and pulmonary embolism (PE), is a significant global healthcare issue and a major cause of unexpected in-hospital deaths. It presents critical challenges for both hospital administrators and clinicians (Wendelboe and Raskob, 2016; ISTH Steering Committee for World Thrombosis Day, 2014; Zhai and Wang, 2015). Since 2024,China’s National Health Commission has prioritized improving VTE prevention rates within its annual National Medical Quality and Safety Improvement Targets. The escalating cancer incidence has further amplified the significance of cancer-associated VTE (CAT), now recognized as a predominant complication among oncology patients (Ma et al., 2019). Notably, VTE confers a 2- to 6-fold elevated mortality risk in this population, establishing thrombosis as a leading cause of cancer-related deaths (Chew et al., 2006; Chew et al., 2007; Sørensen et al., 2000; Kuderer et al., 2008; Martino et al., 2005). Early risk stratification and prophylactic interventions can substantially mitigate hospital-acquired VTE incidence (Li et al., 2018).

Current clinical guidelines from Europe, the United States, and China uniformly recommend low-molecular-weight heparin (LMWH) as first-line therapy for venous thromboembolism prophylaxis in cancer patients (Ma et al., 2019; Streiff et al., 2024; Guidelines Working Committee of the Chinese Society of Clinical Oncology CSCO, 2024; Falanga et al., 2023). In addition to its antithrombotic effects, growing evidence suggests that LMWH may improve cancer survival rates, potentially through pleiotropic mechanisms such as anti-inflammatory and direct antitumor activities. (Wang et al., 2022). Nevertheless, Hemorrhagic complications remain the primary adverse effect of pharmacologic venous thromboembolism (VTE) prevention in this population, presenting a significant challenge for clinicians (Li et al., 2018; Li and Shi, 2020). While existing guidelines acknowledge the elevated bleeding risk associated with anticoagulation in oncology patients, they fail to provide: Validated risk quantification tools, and Dynamic monitoring protocols for anticoagulation-associated hemorrhage (Guidelines Working Committee of the Chinese Society of Clinical Oncology CSCO, 2024). Our preliminary data further demonstrate that:Isolated bleeding risk factors exhibit poor predictive value (AUC <0.65), and The HAS-BLED score,though widely adopted for atrial fibrillation, shows limited discriminative capacity (AUC 0.68, 95% CI 0.62–0.74) in cancer populations (Zhang et al., 2024). This study leverages real-world clinical data to develop a bleeding risk stratification model specifically for CAT patients receiving LMWH anticoagulation. The model integrates oncological characteristics (e.g., tumor type, chemotherapy regimen) with traditional bleeding risk parameters. Its implementation will enable clinicians to: Precisely quantify bleeding probability through dynamic risk scoring, optimize LMWH dosing (prophylactic vs therapeutic) and duration based on individualized risk profiles, and implement personalized thromboprophylaxis strategies that balance VTE prevention with hemorrhage mitigation. Ultimately, this approach seeks to enhance the quality and safety of anticoagulation management by reducing bleeding complications while maintaining therapeutic efficacy, a critical unmet need in oncological practice.

## 2 Materials and methods

### 2.1 Study population

This retrospective cohort study consecutively enrolled 731 adult patients (≥18 years) receiving low-molecular-weight heparin (LMWH) for venous thromboembolism (VTE) prophylaxis or treatment at the Department of Oncology, Chengdu Fifth People’s Hospital.

### 2.2 Group definition and grouping

Grouped according to whether there was a bleeding event in the clinical outcome. Hemorrhage events include: (1) massive hemorrhage (Falanga et al., 2023), which refers to the occurrence of at least one of the following bleeding: blood transfusion of at least 2 units of compressed red blood cells, hemoglobin reduction of 2 g dL-1, bleeding in key parts (intracranial, spinal, intraocular, pericardial, intra-articular, intramuscular compartment syndrome or retroperitoneal) or bleeding leading to death. (2) Clinically relevant non-massive bleeding (Li and Shi, 2020), refers to any bleeding symptoms that do not meet the criteria for massive bleeding but meet at least one of the following criteria: medical intervention; need to be hospitalized or improve the level of care; face-to-face assessment is required if the amount of bleeding exceeds clinical expectations, including bleeding found only by imaging examination. There were 19 patients with bleeding events (bleeding group) and 712 patients without bleeding events (non-bleeding group).

### 2.3 Ethics

The study adhered to the Declaration of Helsinki (2000 edition) and was conducted in accordance with Chinese medical research norms and regulations. The study is a retrospective observational study that does not involve interventional therapy and applies for an exemption from ethics.

Since this was an observational study that did not interfere with clinicians’ decisions or involve the disclosure of patients’ personal privacy information, an exemption from obtaining informed consent from patients or their families was applied for.

### 2.4 Data collection

The primary observation indicators were the occurrence of bleeding events and common bleeding risk factors. Secondary observation indicators included.

• Patient demographic characteristics
• Baseline disease status
• Medical history
• Medication history
• Tumor type
• Coagulation function [prothrombin time (PT), activated partial thrombopl-astin time (APTT), fibrinogen (FIB)]
• Platelet count (PLT)
• Anticoagulation duration
• Thrombosis occurrence
• Clinical scores, etc.

### 2.5 Statistical methods

The data were analyzed using SPSS 25.0 (IBM Corp.). Categorical variables were presented as frequencies (percentages), while continuous variables were expressed as mean ± standard deviation ( $\bar{x}$ ± SD) for normally distributed data or median (interquartile range) [M (IQR)] for non-normally distributed data. Between-group comparisons of categorical variables were performed using χ^2^ tests or Fisher’s exact tests (two-tailed). Variables demonstrating significant associations (P < 0.05) in univariate analyses were entered into a binary logistic regression model to identify independent risk factors for bleeding events, with results reported as adjusted odds ratios (ORs) and 95% confidence intervals (CIs). All statistical tests were two-sided, with P < 0.05 considered statistically significant. For predictive modeling, we employed R software (version 4.4.0; R Foundation) in compliance with TRIPOD 2025 reporting guidelines (Efthimiou et al., 2024), including model calibration and discrimination metrics evaluation.

## 3 Results

### 3.1 General information

A total of 731 patients were included in the model dataset. Preliminary univariate analysis identified the following risk factors influencing bleeding risk in cancer patients using low-molecular-weight heparin (LMWH) for VTE prevention and treatment: body weight, nonsteroidal anti-inflammatory drug (NSAIDs) use, LMWH dosage, prothrombin time (PT), creatinine clearance (Ccr), platelet count (PLT), Padua score, and HAS-BLED bleeding risk score (P < 0.05) (Zhang et al., 2024). The demographic characteristics and related risk factor analysis are presented in Table 1.

**TABLE 1:** Single factor analysis table affecting low molecular weight heparin in the prevention and treatment of bleeding events in patients with VTE tumors.

Observation indicators	Bleeding Group (N = 19)	Non-bleeding Group (N = 712)	χ 2/*t*/*U*	P	Logistic regression analysis		
					*OR*	95%*CI*	*P*
Baseline characteristics							
Male [n (%)]	12 (63.2)	393 (55.2)	0.475	0.491	0.719	0.280–1.847	0.493
Age (years)	61.2 ± 12.3	58.3 ± 10.5	−1.19	0.234	1.029	0.982–1.079	0.232
Weight (kg)	53.2 ± 9.1	59.8 ± 10.09	2.615	0.009	0.933	0.886–0.981	0.007
Tumor classification [n (%)]							
Respiratory System	9 (47.4)	205 (28.8)	3.084	0.079	2.226	0.891–5.558	0.087
Abdominal Solid Organs	1 (5.3)	35 (4.9)	-	1	1.075	0.139–8.282	0.945
Digestive Tract	5 (26.3)	279 (39.2)	1.29	0.256	0.554	0.197–1.556	0.262
Urogenital Tract	3 (15.8)	94 (13.2)	-	0.73	1.233	0.352–4.311	0.743
Other	1 (5.3)	99 (13.9)	-	0.496	0.344	0.45–2.606	0.302
Baseline disease status [n (%)]							
History of Hypertension	5 (26.3)	223 (31.3)	0.216	0.642	0.783	0.279–2.201	0.643
Uncontrolled Hypertension	2 (10.5)	36 (5.1)	-	0.259	2.209	0.491–9.931	0.301
History of Diabetes	3 (15.8)	95 (13.3)	-	0.732	1.218	0.348–4.258	0.758
Active Peptic Ulcer	1 (5.3)	10 (1.4)	-	0.253	3.9	0.474–32.110	0.206
History of Bleeding in 3 Months	1 (5.3)	20 (2.8)	-	0.429	1.922	0.244–15.114	0.535
History of Major Bleeding or Acute Stroke in 1 Month	0 (0)	2 (0.3)	-	1	0	-	-
Long-Term Alcohol Use	3 (15.8)	136 (19.1)	-	1	0.794	0.228–2.764	0.717
Central Venous Catheter	10 (52.6)	488 (68.7)	2.216	0.137	0.505	0.203–1.261	0.144
Medication use [n (%)]							
NSAIDs Use	15 (78.9)	200 (28.1)	23.055	<0.001	9.6	3.148–29.275	<0.001
Antiplatelet Drugs	1 (5.3)	30 (4.2)	-	0.566	1.263	0.163–9.777	0.823
Oral Anticoagulants	2 (10.5)	60 (8.4)	-	0.671	1.278	0.288–5.666	0.746
LMWH Dosage (IU/kg)	100 (90)	70 (18)	3284.5	<0.001	1.015	1.007–1.024	<0.001
Duration of Medication (days)	4 (4)	5 (6)	6248.5	0.568	0.993	0.914–1.078	0.863
Laboratory indicators							
PT(s)	12 (2.6)	11.4 (1.3)	4266.5	0.006	1.194	1.081–1.318	<0.001
APTT(s)	31.3 (5.7)	31.4 (4.4)	6424	0.708	1.022	0.921–1.133	0.681
FIB(g/L)	3.64 ± 1.29	3.65 ± 1.0	0.055	0.956	0.987	0.627–1.556	0.956
Ccr(ml/min)	67.95 ± 30.58	93.64 ± 33.85	3.273	<0.001	0.971	0.955–0.988	<0.001
Ccr categories [n (%)]							
Ccr<30 mL min^–1^	2 (10.5)	7 (1.0)	-	0.021	11.849	2.291–61.293	0.003
30≤Ccr≤60 mL min^–1^	5 (26.3)	299 (42.0)	1.873	0.171	0.493	0.176–1.384	0.180
60<Ccr≤90 mL min^–1^	13 (68.4)	388 (54.5)	1.449	0.229	1.809	0.680–4.814	0.235
PLT/(×10^9^/L)	147 ± 101	197 ± 85	2.471	0.014	0.991	0.983–0.998	0.011
PLT<100 (×10^9^/L)	9 (47.4)	65 (9.1)	29.742	<0.001	8.958	3.514–22.841	<0.001
Clinical scores							
Padua Score	7 (4)	4 (1)	1877.5	<0.001	2.042	1.643–2.537	<0.001
HAS-BLED Score	3 (2)	1 (2)	2119.5	<0.001	2.4	1.752–3.287	<0.001
HAS-BLED ≥3 [n (%)]	14 (73.7)	82 (11.5)	62.695	<0.001	21.512	7.553–61.273	<0.001

### 3.2 Model variable screening

From Tab1, it was observed that factors such as nonsteroidal anti-inflammatory drugs (NSAIDs), prothrombin time (PT), and creatinine clearance (Ccr) were already included in the HAS-BLED bleeding risk score. Additionally, the Padua score, primarily used to assess the risk of VTE occurrence, was excluded based on clinical relevance. Therefore, the preliminary variables selected for the logistic regression model were body weight, low-molecular-weight heparin (LMWH) dosage, platelet count, and the HAS-BLED bleeding risk score. The Box-Tidwell test indicated that continuous variables (body weight, LMWH dosage, and HAS-BLED score) had a linear relationship with bleeding events, while platelet count exhibited a nonlinear relationship (Zhang et al., 2024). To meet the assumptions of the logistic regression model, platelet count was transformed into a binary categorical variable (classified as platelet count <100 × 10^9^/L or ≥100 × 10^9^/L). These four variables were then incorporated into the logistic regression model, and backward stepwise regression analysis was performed. The final model included LMWH dosage, platelet count, and the HAS-BLED score as evaluation factors. The relevant data for the model are presented in Table 2.

**TABLE 2:** Variables and related parameters included in the model.

Variable	Univariate analysis					Multivariate analysis				
	β	S.E	Z	*P*	OR (95%CI)	β	S.E	Z	*P*	OR (95%CI)
PLT<100*10^9^L^–1^, n (%)										
0					1.00(Reference)					1.00(Reference)
1	2.33	0.56	4.18	<0.001	10.27 (3.45–30.63)	2.27	0.62	3.69	<0.001	9.72 (2.90–32.58)
HAS-BLED Score	0.84	0.19	4.43	<0.001	2.31 (1.60–3.35)	0.79	0.21	3.75	<0.001	2.21 (1.46–3.34)
LMWH Dosage	0.02	0	3.34	<0.001	1.02 (1.01–1.03)	0.01	0.01	2.09	0.037	1.01 (1.01–1.02)

Abbreviations: OR, odds ratio; CI, confidence interval.

### 3.3 Internal validation results of the model

In accordance with the guidelines for clinical prediction model distribution (Efthimiou et al., 2024), the dataset was randomly divided into a training set (510 cases) and a validation set (221 cases) at a ratio of 7:3. Both sets included the following variables: HAS-BLED bleeding risk score, low-molecular-weight heparin (LMWH) dosage, platelet count <100 × 10^9^/L, and bleeding events. No statistically significantdifferences were observed in these variables between the two sets (P > 0.05). Detailed results are presented in Table 3.

**TABLE 3:** Variable difference analysis between the training and validation sets.

Category	Dataset (n = 731)	Validation set (n = 221)	Training set (n = 510)	Test value	P value
HAS-BLED Score, M (QR)	1.00 (2)	1.00 (2)	1.00 (2)	Z = −0.23	0.815
LMWH Dosage, M (QR)	70.18 (61.54, 81.63)	70.80 (64.00, 83.33)	70.18 (61.54, 80.00)	Z = −1.09	0.278
PLT <100 × 10^9^/L, n (%)				χ^2^ = 0.03	0.867
0	657 (89.88%)	198 (89.59%)	459 (90.00%)		
1	74 (10.12%)	23 (10.41%)	51 (10.00%)		
Bleeding Events, n (%)				χ^2^ = 0.14	0.706
0	712 (97.40%)	216 (97.74%)	496 (97.25%)		

Abbreviations: M, median; QR, interquartile range; Z, Mann-Whitney Test; χ^2^, Chi-square Test.

### 3.4 Model prediction performance evaluation

The predictive performance of the model for bleeding events was evaluated using the Receiver Operating Characteristic (ROC) curve. The results demonstrated excellent predictive ability, with the following Area Under the Curve (AUC) values and 95% confidence intervals (CI).

• Training Set: AUC = 0.90 (95% CI: 0.82–0.97)
• Validation Set: AUC = 0.96 (95% CI: 0.93–0.99)

**FIGURE 1:**
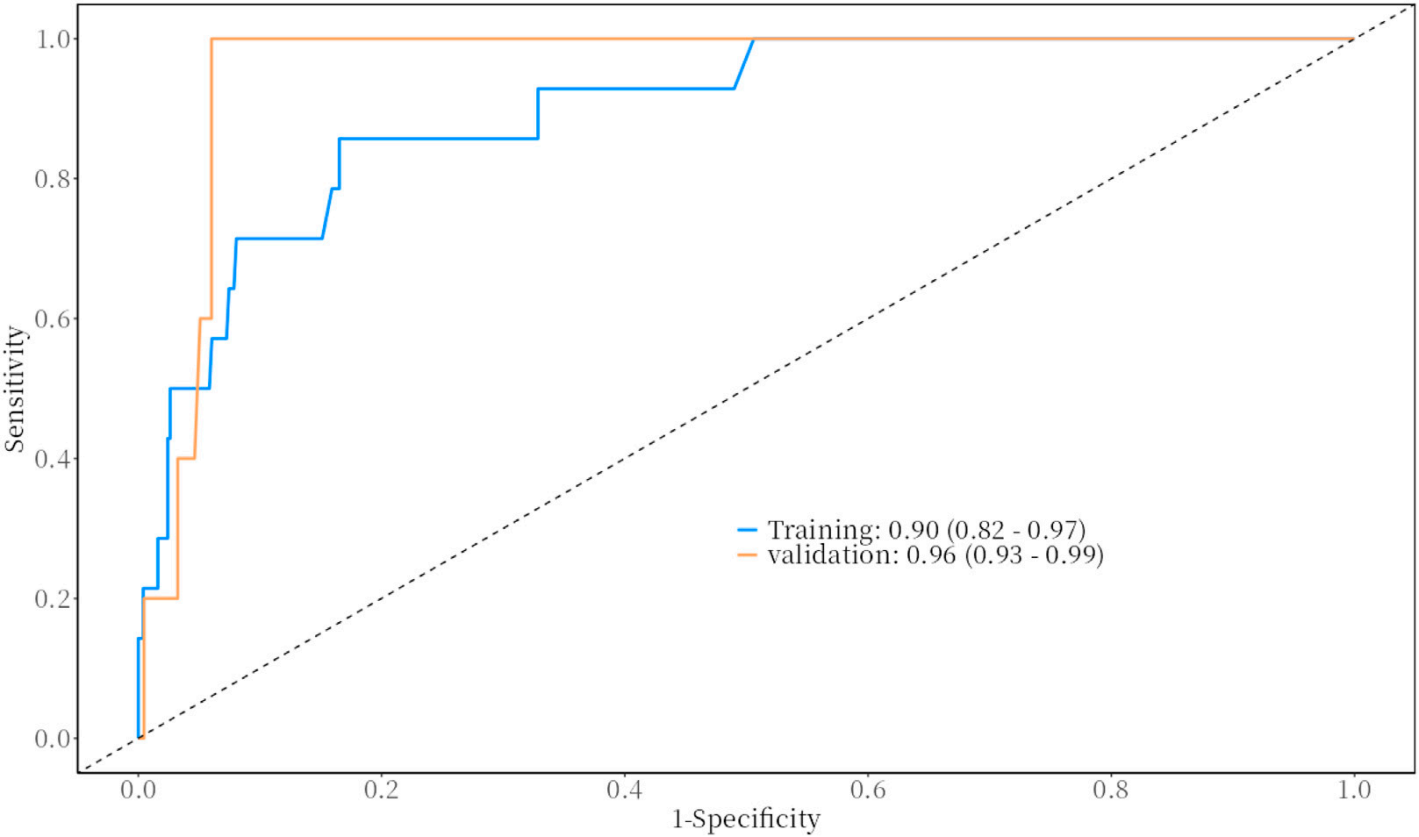
The ROC curve of the training and validation.

These results indicate that the model has strong discriminative power for predicting bleeding events in both the training and validation datasets. For detailed visualization, please refer to Figure 1.

### 3.5 Model discrimination evaluation

**FIGURE 2:**
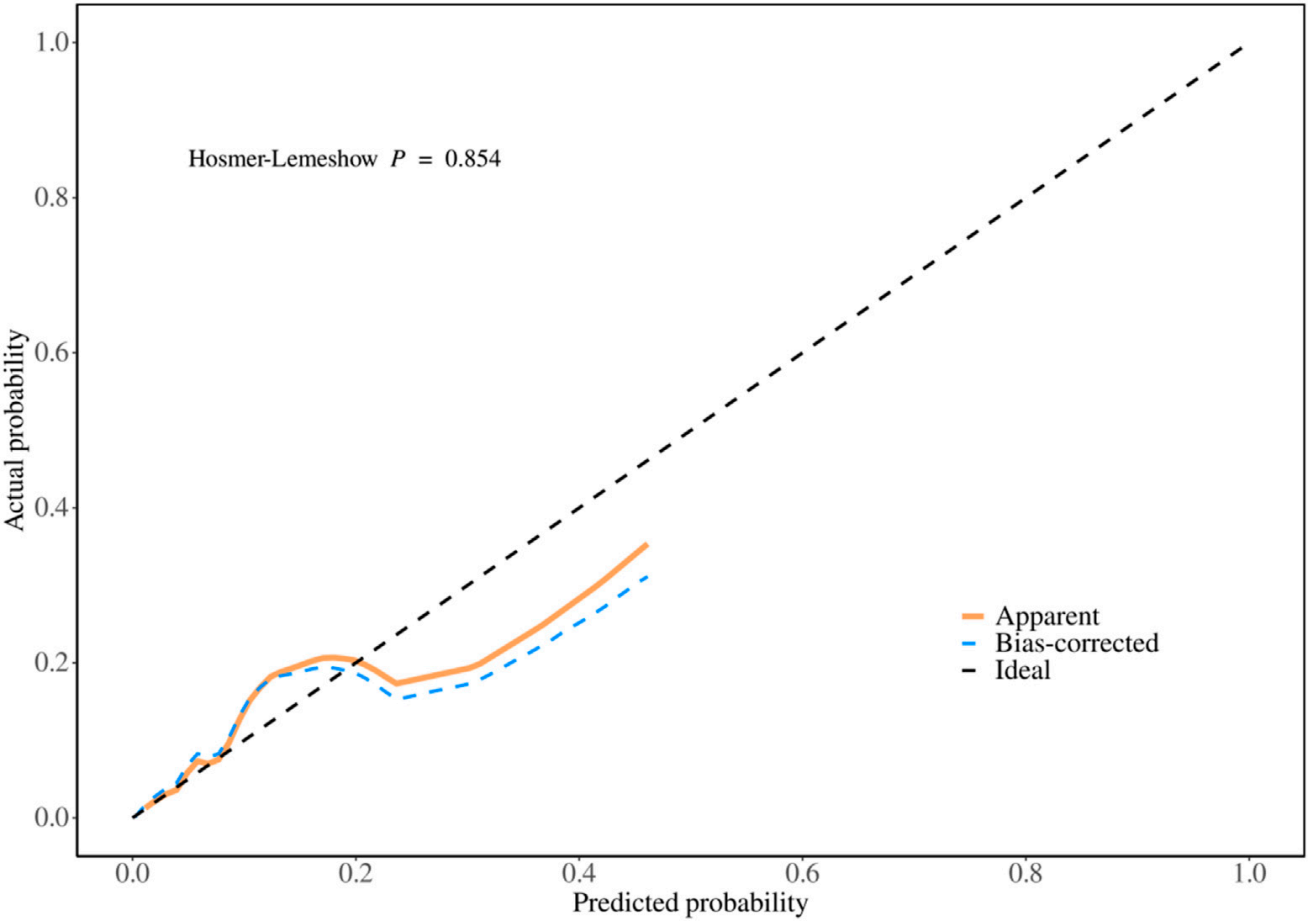
Model calibration curve.

The discrimination ability of the model for bleeding events was assessed by plotting a calibration curve using the R language (Figure 2). As shown in the figure, the actual curve of the model closely aligns with the calibration curve, indicating a high level of consistency between predicted and observed values.

To further validate the discrimination ability, the Hosmer-Lemeshow test was performed, yielding the following result.

• Hosmer-Lemeshow Test P-value: 0.854 (>0.05)

This result suggests that the model demonstrates good discrimination ability in distinguishing between bleeding and non-bleeding events, with no significant difference between predicted and actual outcomes. The model exhibits high calibration and reliability. Please refer to Figure 2.

### 3.6 Model decision-making ability evaluation

**FIGURE 3:**
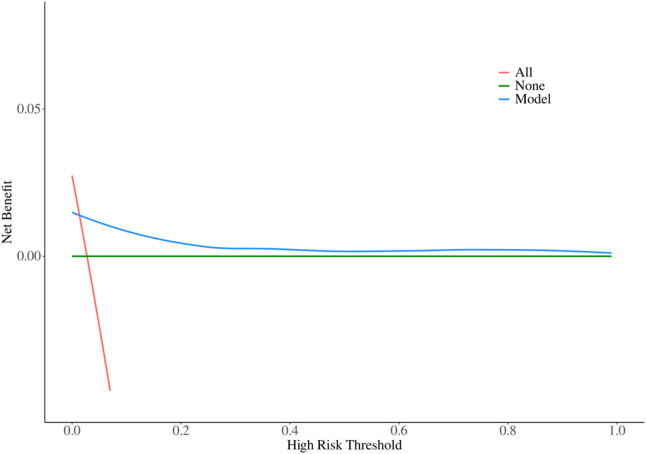
Model DCA curves.

The decision-making ability of the model for bleeding events was evaluated by plotting a Decision Curve Analysis (DCA) curve using the R language (Figure 3). As shown in the figure, within the probability threshold range of 0.1–1, the model curve consistently lies above both the positive curve (treating all patients as positive) and the negative curve (treating all patients as negative). This indicates that the model has excellent decision-making ability and provides significant clinical net benefit.

These results suggest that the model has substantial clinical value in predicting bleeding events, offering reliable decision support for clinicians to optimize treatment strategies and improve patient outcomes. For detailed visualization, please refer to Figure 3.

### 3.7 Model diagnostic ability evaluation

The diagnostic ability of the model was comprehensively analyzed using data from the training and validation sets, focusing on its predictive power, accuracy, sensitivity, specificity, positive predictive value (PPV), and negative predictive value (NPV). The results indicate that both sensitivity and specificity are high, demonstrating excellent diagnostic performance and a low probability of missed diagnoses. The diagnostic performance of the model is detailed in Table 4.

**TABLE 4:** Evaluation table of model diagnostic ability.

Dataset	AUC (95%CI)	Accuracy (95%CI)	Sensitivity (95%CI)	Specificity (95%CI)	PPV (95%CI)	NPV (95%CI)	cut off
Training Set (n = 510)	0.90 (0.82–0.97)	0.83 (0.80–0.86)	0.83 (0.80–0.87)	0.79 (0.57–1.00)	0.99 (0.98–1.00)	0.12 (0.05–0.18)	0.028
Validation Set (n = 221)	0.96 (0.93–0.99)	0.85 (0.80–0.89)	0.85 (0.80–0.90)	1.00 (1.00 - 1.00)	1.00 (1.00 - 1.00)	0.13 (0.02–0.24)	0.028

### 3.8 Model clinical scoring table

Finally, a nomogram was created using the R language to facilitate clinical scoring. The scoring system is designed as follows.

• Platelet Count (PLT):
  • PLT <100 × 10^9^/L is assigned a score of 1, corresponding to 48 points.
  • PLT ≥100 × 10^9^/L is assigned a score of 0, corresponding to 0 points.
• HAS-BLED Score:
  • Scores range from 0 to 6, corresponding to 0 to 100 points.
• LMWH Dosage:
  • Dosages range from 20 to 260, corresponding to 0 to 100 points.

**FIGURE 4:**
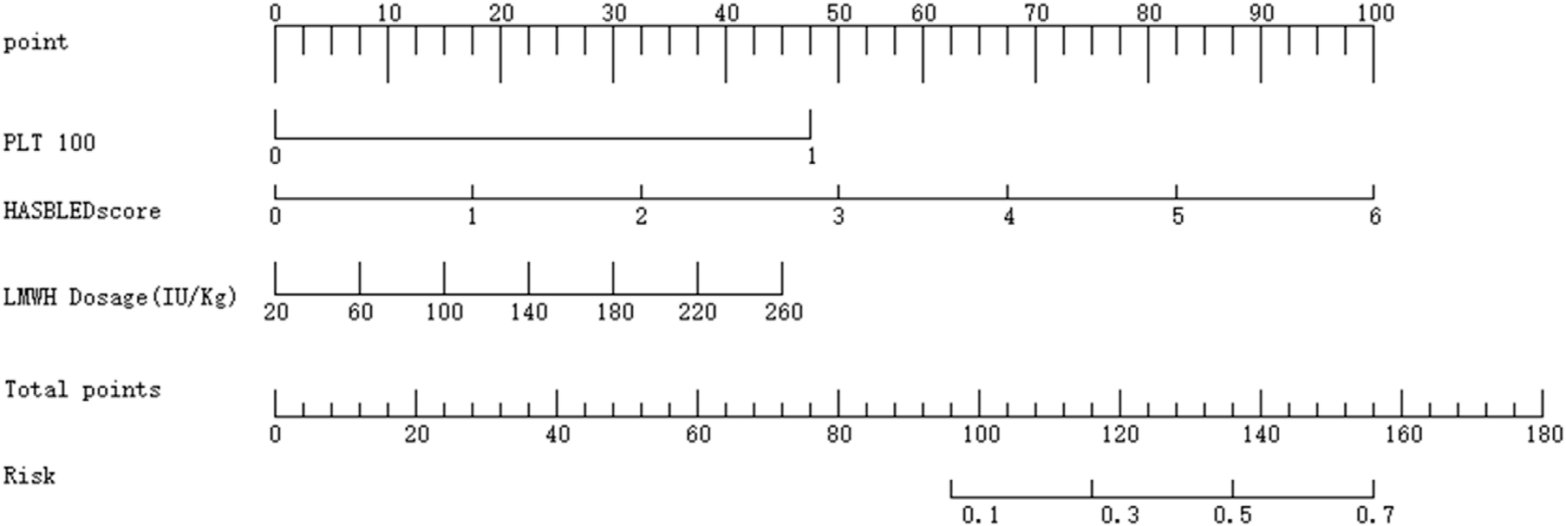
Model clinical score scale.

The total score is calculated by summing the points from the three variables. The total score is then mapped to a risk scale, which provides the predicted probability of bleeding risk. For detailed visualization, please refer to Figure 4.

## 4 Discussion

Low-molecular-weight heparin is essential for anticoagulation in cancer patients with venous thromboembolism. The primary risk associated with anticoagulation therapy is the occurrence of bleeding events during treatment. The 2024 Guidelines for VTE Prevention and Treatment in Cancer Patients highlight that there is no specific bleeding risk prediction model tailored for cancer patients, and existing models such as the IMPROVE model and the RIETE scoring system have not reached a consensus for use in oncology patients (Guidelines Working Committee of the Chinese Society of Clinical Oncology CSCO, 2024). The IMPROVE score (Decousus et al., 2010), primarily used for assessing bleeding risk during VTE prophylaxis, includes factors such as age, sex, malignancy, prior bleeding history, platelet count, and liver and kidney function. It is designed for acutely ill medical inpatients. However, its predictive ability for bleeding risk in other high-risk VTE populations, such as orthopedic surgery, gynecological surgery, and cancer patients, remains to be validated (Lin and Zhang, 2019). Additionally, the IMPROVE model targets all VTE patients and includes various anticoagulants, not just LMWH, which differs from the LMWH-focused cancer patient population in this study. The RIETE scoring system (Ruíz-Giméne et al., 2008) focuses on identifying VTE patients at higher risk of bleeding, incorporating factors such as age, malignancy, recent bleeding history, anemia, creatinine levels, and significant pulmonary embolism. However, it is not suitable for evaluating the timing and dosage adjustments of LMWH, as proposed in this study. Moreover, the RIETE system lacks data on model discrimination and consistency, and external validation has shown poor predictive performance, particularly for acute VTE patients on conventional anticoagulation therapy (Lin and Zhang, 2019; Klok et al., 2016). The HAS-BLED score, widely used for bleeding risk assessment in non-valvular atrial fibrillation patients, includes factors such as hypertension, abnormal renal and liver function, prior bleeding history, and alcohol or drug use (Lip et al., 2010). However, preliminary studies have shown that it does not perform well in CAT patients, limiting its applicability in this context.

A study validating eight scoring models found that the HAS-BLED model demonstrated the highest sensitivity and negative predictive value but also highlighted the complexity of balancing risks and benefits in conditions such as malignancy, which predisposes patients to recurrent thromboembolic events and major bleeding complications. In contrast, the RIETE score showed poor predictive validity (Riva et al., 2014). Therefore, based on clinical practice and literature review, this study developed a bleeding risk prediction model specifically for cancer patients undergoing LMWH anticoagulation for VTE. The model builds on the HAS-BLED score by incorporating two additional variables: LMWH dosage and platelet count. These additions align with the clinical presentation of cancer patients on LMWH, as LMWH dosage is a critical determinant of bleeding risk, and platelet count plays a key role in coagulation and is significantly influenced by LMWH. Preliminary studies identified several bleeding risk factors in cancer patients using LMWH for VTE prevention, including body weight, nonsteroidal anti-inflammatory drug use, LMWH dosage, PT, creatinine clearance, platelet count, Padua score, and HAS-BLED score. However, their individual predictive abilities were suboptimal (Zhang et al., 2024). Given that some of these factors are already included in the HAS-BLED score, this study developed an improved model by adding LMWH dosage and platelet count to the HAS-BLED score. The model’s predictive performance (ROC-AUC: 0.90 vs 0.843 for the HAS-BLED score) significantly improved to an excellent level (Zhang et al., 2024). The model’s discrimination, decision-making, and diagnostic abilities were further evaluated using calibration curves, decision curve analysis (DCA), and diagnostic performance metrics, all of which demonstrated favorable results. Finally, a nomogram was created to facilitate clinical scoring. This model quantifies bleeding risk based on different LMWH dosages, enabling healthcare providers to individualize LMWH therapy rather than relying on fixed doses as per drug guidelines. It also assesses LMWH dosage adjustments based on baseline HAS-BLED scores and platelet counts, allowing for dynamic evaluation and modification of anticoagulation therapy during treatment.

### 4.1 Limitations

This study has several limitations.

1. Inclusion of High-Risk Factors: Due to the high-risk nature of certain factors mentioned in the guidelines, standardized anticoagulation was not administered in clinical practice, resulting in insufficient case collection for analysis. This may introduce errors in predicting anticoagulation risks for such patients.
2. Single-Center Data: The model was developed and internally validated using single-center data, and its real-world applicability may differ from internal evaluation metrics. External validation and multicenter data are needed for further refinement.
3. Specific Population: The model is tailored for cancer patients using LMWH for VTE prevention and may not apply to those using other anticoagulants or non-cancer VTE patients on LMWH.

## 5 Conclusion

This study successfully established a drug anticoagulation bleeding risk prediction model for tumor patients. The HAS-BLED bleeding risk score, low molecular weight heparin usage, and platelet count contained in the model may help predict the bleeding risk of low molecular weight heparin anticoagulation in tumor patients. The model is based on retrospective single-center data, and the internal evaluation indicators of the model are excellent. Nevertheless, the differences in the background of patients from different institutions or different ethnic groups have not been fully evaluated. We need to further verify the model through a larger sample size and multi-center institutions. It is hoped that the model can be applied to clinical practice to help medical staff to individually evaluate the timing of starting and stopping the use of low molecular weight heparin anticoagulation in tumor patients and to minimize the occurrence of bleeding events.

## Data Availability

The original contributions presented in the study are included in the article/Supplementary Material, further inquiries can be directed to the corresponding author.
